# Sexual function assessment in patients with SAPHO syndrome: a cross-sectional study

**DOI:** 10.1186/s13023-023-02826-y

**Published:** 2023-07-27

**Authors:** Chen Li, Haixu Jiang, Yunan Zhang, Guangrui Huang

**Affiliations:** 1 Department of Rheumatology, Fangshan Hospital, Beijing University of Chinese Medicine, Beijing, 102401 China; 2 Department of Traditional Chinese Medicine, Peking Union Medical College Hospital, Peking Union Medical College and Chinese Academy of Medical Sciences, Beijing, 100730 China; 3 School of Chinese Materia, Beijing University of Chinese Medicine, Beijing, 102488 China; 4 School of Life Sciences, Beijing University of Chinese Medicine, Beijing, 102488 China

**Keywords:** SAPHO syndrome, Sexual dysfunction, Autoimmune disease, Quality of sexual life, Mental state

## Abstract

**Introduction:**

SAPHO syndrome is a group of special syndromes characterized by synovitis, acne, pustulosis, hyperostosis and osteitis. Skin lesions and joint damage are the main clinical manifestations. Among them, females mostly present with palm toe pustulosis, while males have severe acne as the main external manifestation. The bone and joint damage characterized by bone hypertrophy and osteitis is the core manifestation of SAPHO and affects all parts of the body. SAPHO syndrome causes great physical and mental suffering to patients, and it also brings a huge financial burden to the family. The purpose of this study is to explore the impact of SAPHO on the quality of sexual life of patients.

**Methods:**

We screened and included 249 SAPHO patients (169 women and 80 men) from Peking Union Medical College Hospital (Beijing, China). First, we recorded the basic situation of the patient through questionnaires (including gender, age, SAPHO duration, BMI, smoking, drinking, marital status, educational level, occupational status and work status.). Then, the patient needed to fill in the Short Form-36 quality of life questionnaire (SF-36 QoL) to record the quality of life. For Sexual dysfunction (SD), female patients needed to fill in the Female Sexual Function Index (FSFI) to assess the quality of sexual life; while the International Index of Erectile Function (IIEF) was used to assess the SD of male patients. At the same time, we used self-esteem and relationship questionnaire (SEAR) to analyze the psychological state of SAPHO patients. Finally, we performed statistical analysis on the data obtained, and then explored the connection between SAPHO and SD.

**Results:**

In this cross-sectional study, a total of 249 patients completed the questionnaire and constituted the study population. We found that among 169 female patients, 124 patients had FSD (73.4%); while 45 patients did not have FSD (26.6%); and among 80 male patients, 45 (56.3%) had ED; However, 35 patients did not have ED (43.7%). The results of the quality of life and mental state assessment showed that female patients with SD showed lower scores in terms of mental state. Among all male participants, we found no significant difference in quality of life and mental state among participants with or without SD. In addition, there was no significant difference in the duration of SAPHO between female and male participants with or without SD.

**Conclusion:**

This study is the first to evaluate the SD of SAPHO patients. The incidence of SD in female SAPHO patients is higher than that in male patients; the cause of female SD may be mainly psychological factors. These results prove that it is particularly important to focus on regulating their psychological state while diagnosing and treating SAPHO patients in clinical practice.

## Introduction

In 1987, Chamot, a French rheumatologist, described a special disease process characterized by skin lesions and joint damage co-existing in the affected individuals, and named it SAPHO syndrome with the initials of the words of synovitis, acne, pustulosis, hypertrophy and osteitis [1]. SAPHO and the disease spectrum it represents are considered rare. Due to the lag of its diagnosis and the uncertainty of clinical disease manifestations, its exact prevalence is still elusive [2]. According to published studies, there are reports of SAPHO diseases worldwide. The annual prevalence rate of European whites is less than 1/10,000, while the annual prevalence rate of Japanese is less than 0.00144/100,000. However, due to insufficient knowledge of the disease in China, there is no report on the exact incidence of SAPHO [3]. As of 2020, 1,000 SAPHO cases have been reported worldwide [4, 5]. The pathogenesis of SAPHO syndrome is unclear, and may be related to immune dysfunction [6], infection [7] and genetic susceptibility [8].There is no consensus on SAPHO’s treatment plan. The current main strategy is to ameliorate clinical symptoms and improve the quality of life of patients in the long time. The main therapeutic drugs include: non-steroidal anti-inflammatory drugs (NSAIDs), Disease-modifying antirheumatic drugs (DMARDs), Corticosteroids, Bisphosphonates and so on. TNF-α antagonists are also increasingly used in the treatment of SAPHO, and have achieved good clinical effects. Historically, SAPHO mainly affects young and middle-aged people [2], and the disease recurs [9]. So it may impair the quality of sexual life of patients.

Sexual dysfunction (SD) is characterized by desire, arousal, orgasm and pain, and its pathogenesis is more complicated, including a variety of physical and psychological factors. Historically, sexual dysfunction is attributed to the psychological field [10]. Sexual dysfunction is one of the common symptoms of Behçet’s disease (BD) [11]. But the data on SD occurrence in this particular group of patients is limited. As far as we know, the assessment of SAPHO patients’ anxiety, work status and quality of life has been the subject of numerous studies, no assessment of sexual dysfunction in patients with SAPHO syndrome has been carried out. This study is the first to assess sexual function of SAPHO syndrome patients and its purpose was to determine the prevalence of SD and to analyze the potential risk factors for SD.

## Methods

### Patient enrolment and study design

This study was approved by the ethics committee of PUMCH (Identifier: ZS-944) (Beijing, China). This study was conducted using a single-centre, cross-sectional design. The inclusion criteria included a diagnosis of SAPHO syndrome according to the diagnosis criteria proposed by Kahn [12] in 2003, and symptoms like bone pain persisting in the most recent one month. 18–60 years old, those who have had sex in the past 4 weeks. After obtaining the informed consent of PUMCH participants, they were asked to complete a questionnaire about the quality of sexual life. This study was funded by the National Key Research and Development Program of China (2016YFC0901500) and CAMS Innovation Fund for Medical Sciences (CIFMS) [2017-I2M-3-001].

The questionnaire completed by the patient includes demographic, quality of life, quality of sexual life data and mental state. Demographic and clinical data include gender, age, SAPHO duration, BMI, smoking, drinking, marital status, educational level, occupational status and work status. The disease related quality of life was measured with the help of the Short Form-36 quality of life questionnaire (SF-36 QoL). The SF-36 QoL questionnaire mainly involves the following areas: Physical-Functioning, role-limit-physical, role-limit-emotional, energy-fatigue, emotional-well-being, social-functioning, pain and general-health. The SF-36 QoL scale was in the supplementary material. The main variable “sexual dysfunction” in this study was evaluated differently in men and women. The Female Sexual Function Index (FSFI) has been validated as a reliable questionnaire to assess female sexual function in general Chinese population [13]. The FSFI scale involves six female sexual areas: Desire, Arousal, Lubrication, Orgasm, Satisfaction and Pain. It mainly contains 19 questions to investigate sexual problems in the past 4 weeks. The first two questions are scored 1–5, and the remaining questions are scored 0–5. A total score of less than 23 is defined as the critical value for distinguishing FSD. (FSD was defined as “lack or reduced sexual interest or excitement”). The FSFI scale was in the supplementary material. The International Index of Erectile Function (IIEF) is often used to assess the quality of sexual life of male patients [14]. Male SD is represented by erectile dysfunction (ED). The IIEF scale involves five sexual domains of men: Erectile function, Orgasmic function, Sexual desire, Intercourse satisfaction and Overall satisfaction. This is an erectile dysfunction questionnaire containing 15 questions. The first 9 questions are scored 0–5, and the rest are scored 1–5. Scores below 25 are considered ED. (The European Urological Association defined erectile dysfunction as “continuous failure to achieve and maintain sufficient erectile ability to obtain satisfactory sexual behavior.) The IIEF scale was in the supplementary material. At the same time, the self-esteem and relationship questionnaire (SEAR) questionnaire was used to assess the mental state of participants. The SEAR questionnaire is a questionnaire containing 14 questions. It explains the psychological state of participants in two major aspects: Sexual relationship (Q1-Q8) and Confidence (Q9-Q14). Among them, Confidence (Q9-Q14) includes two small aspects: Self-esteem (Q9- Q12) and Overall relationship (Q13-Q14). The SEAR scale was in the supplementary material.

### Statistical analysis

The data are expressed as the mean, standard deviation, number and percentage of categorical variables of continuous variables. Continuous variables used t test or Mann-Whitney Utest, categorical variables used chi-square test or Fisher’s exact test. Statistical analysis was performed with SPSS (version 25.0), and a two-sided P value of less than 0.05 was considered statistically significant.

## Results

### Epidemiological characteristics of the participants in this study

249 patients completed all of the questionnaires and constituted the study population. Of the 249 patients in this cross-sectional study, 80 were males and 169 were females. The age of the patients is between 18 and 59 years old, and most of them are between 30 and 59 years old. Regarding BMI, the BMI of most patients was between 18.5 and 28. Only a small percentage of patients have the habit of smoking and drinking. Among all the participants, the vast majority were already married. The education level of the patients was evenly distributed from low to high. In the work status survey, “Employed” and “Retired / housewives” each account for half. As for the most important part-SAPHO duration: 52.6% of patients with SAPHO disease progression less than 5 years; 33.7% of patients with 5–10 years; and 13.7% of patients with more than 10 years. Demographic data from these couples was presented in Table 1.

**Table 1:** Epidemiological data of participants in this study (n = 249)

Epidemiological characteristics		Number	Percentage (%)
Gender			
	Male	80	32.1
	Female	169	67.9
Age, years			
	18 ~ 29	18	7.2
	30 ~ 39	87	34.9
	40 ~ 49	75	30.1
	50 ~ 59	69	27.7
SAPHO duration, years			
	< 5	131	52.6
	5 ≤ to < 10	84	33.7
	≥ 10	34	13.7
BMI, kg/m^2^			
	< 18.5	9	3.6
	18.5 ≤ to < 24	123	49.4
	24 ≤ to < 28	81	32.5
	≥ 28	36	14.5
Smoking			
	YES	72	28.9
	NO	177	71.1
Drinking			
	YES	17	6.8
	NO	232	93.2
Marital status			
	Married	232	93.5
	Single / Divorced / Widowed	16	6.5
Educational level			
	Primary or junior high school	62	24.9
	Senior high school	85	34.1
	University or college	72	28.9
	Master’s or further education	30	12.0
Occupational status			
	Brain work	159	63.9
	Physical work	72	28.9
	Brain and physical	5	2.0
	Homemaker	13	5.2
Work status			
	Employed	129	51.8
	Retired / housewives	120	48.2

### Results of sexual function assessment using FSFI (for female) and IIEF (for male)

Among all the participants in this cross-sectional study, whether they suffered from SD was described in Table 2. The FSFI scale showed that among 169 female patients, 124 patients had FSD (73.4%); while 45 patients had no FSD (26.6%). The IIEF scale showed that among 80 male patients, 45 patients suffered from ED (56.3%); while 35 patients did not have ED (43.7%). In addition, in the FSFI scale, the scores of FSD and nFSD patients in the scale were significantly different (p < 0.01). The IIEF scale also described similar results (p < 0.01).

**Table 2:** Results of sexual function assessment using FSFI (for female) and IIEF (for male)

FSFI: Female Sexual Function Index								
	Total (n = 169)			FSD (124, 73.4%)		nFSD (45, 26.6%)		p
	Mean	SD	Range	Mean	SD	Mean	SD	
Desire	2.52	1.02	1.2 ~ 6	2.12	0.77	3.61	0.81	< 0.01*
Arousal	2.22	1.62	0 ~ 6	1.5	1.2	4.21	0.7	< 0.01*
Lubrication	3.08	2.26	0 ~ 6	2.2	2	5.51	0.44	< 0.01*
Orgasm	2.54	1.96	0 ~ 6	1.76	1.66	4.69	0.66	< 0.01*
Satisfaction	3.29	1.28	0.8 ~ 6	2.79	1.07	4.67	0.61	< 0.01*
Pain	2.27	1.55	0 ~ 5.2	1.96	1.67	3.15	0.49	< 0.01*
Full scale score	15.93	8.63	2.3 ~ 31.2	12.33	7.15	25.85	2.1	< 0.01*
IIEF: International Index of Erectile Function								
	Total (n = 80)			ED (45, 56.3%)		nED (35, 43.7%)		p
	Mean	SD	Range	Mean	SD	Mean	SD	
Erectile function	20.76	9.36	1 ~ 30	14.69	8.34	28.57	1.48	< 0.01*
Orgasmic function	6.85	3.78	0 ~ 10	4.73	3.82	9.57	0.85	< 0.01*
Sexual desire	6.45	2.38	2 ~ 10	5.11	2.13	8.17	1.36	< 0.01*
Intercourse satisfaction	7.34	4.42	0 ~ 14	4.87	4.1	10.51	2.27	< 0.01*
Overall satisfaction	6.70	1.85	2 ~ 10	5.82	1.66	7.83	1.44	< 0.01*
Full scare score	48.10	19.88	5 ~ 74	35.22	17.2	64.66	5.84	< 0.01*

### Comparison of clinical characteristics between participants with and without sexual dysfunction

SF-36 QoL and SEAR were used to analyze the clinical status of all participants with or without SD. The specific data analysis was illustrated in Tables 3 and 4. Among the female participants in this study, there were no significant differences in BMI, age, smoking, drinking, marital status, educational level, occupational status and work status among female participants with or without SD. In the SF-36 QoL scale analysis, female participants with or without SD showed no significant differences in physical-functioning, role-limit-physical, role-limit-emotional, social-functioning pain and general-health; but participants with SD showed lower scores in energy-fatigue (p = 0.01) and emotional-well-being (p = 0.02). In the SEAR scale survey, compared with normal participants, participants with SD showed lower scores in the areas of Sexual relationship, Self-esteem, Confidence and Overall relationship (p < 0.001). Among all male participants, we found that there were no significant differences in demographic, quality of life (SF-36 QoL) and mental state (SEAR) among participants with or without SD. In addition, there was no significant difference in SAPHO duration between female and male participants, with or without SD.

**Table 3:** Comparison of clinical characteristics between females with and without sexual dysfunction

	Female sexual dysfunction	No female sexual dysfunction	p
Married (n, %)	118, 95.2	43, 95.6	1
Current smoker (n, %)	16, 12.9	11, 24.4	0.07
Current drinker (n, %)	0, 0	3, 6.7	0.018*
Higher education (n, %)	55, 44.4	15, 33.3	0.199
Mental work dominant (n, %)	78, 62.9	33, 73.3	0.207
Employeed (n, %)	59, 47.6	24, 53.3	0.508
Age, years (mean, SD)	43.88, 9.86	42.44, 8.39	0.39
SAPHO duration, years (mean, SD)	5.57, 4.94	5.49, 4.90	0.92
BMI, kg/m^2^ (mean, SD)	23.58, 3.61	24.34, 6.56	0.34
QoL PF (mean, SD)	77.26, 17.81	82.11, 17.56	0.712
QoL RP (mean, SD)	42.74, 43.68	51.67, 45.97	0.785
QoL RE (mean, SD)	42.74, 41.98	51.11, 47.46	0.746
QoL VT (mean, SD)	57.34, 21.23	66.67, 19.31	0.01*
QoL MH (mean, SD)	61.26, 19.22	68.89, 15.40	0.02*
QoL SF (mean, SD)	69.76, 23.38	77.50, 21.09	0.726
QoL BP (mean, SD)	64.92, 18.85	69.00, 22.39	0.24
QoL GH (mean, SD)	48.55, 18.78	54.44, 20.70	0.08
Sexual relationship (mean, SD)	31.83, 20.16	71.88, 17.00	0.00***
Self-esteem (mean, SD)	55.65, 25.20	83.89, 14.63	0.00***
Confidence (mean, SD)	54.10, 24.11	84.35, 14.19	0.00***
Overall relationship (mean, SD)	51.01, 31.82	85.28, 16.05	0.00***

**Table 4:** Comparison of clinical characteristics between males with and without sexual dysfunction

	Erectile dysfunction	No erectile dysfunction	p
Married (n, %)	40, 88.9	32, 91.4	1
Current smoker (n, %)	24, 53.3	21, 60	0.551
Current drinker (n, %)	5, 11.1	9, 25.7	0.88
Higher education (n, %)	19, 42.2	13, 37.1	0.645
Mental work dominant (n, %)	31, 68.9	22, 62.9	0.571
Employeed (n, %)	27, 60	19, 20.1	0.608
Age, years (mean, SD)	39.44, 7.72	41.29, 8.67	0.32
SAPHO duration, years (mean, SD)	4.79, 3.67	5.69, 5.00	0.36
BMI, kg/m^2^ (mean, SD)	26.02, 3.78	24.72, 3.54	0.12
QoL PF (mean, SD)	81.00,21.84	86.71, 12.54	0.059
QoL RP (mean, SD)	57.22, 45.11	58.57, 45.35	0.31
QoL RE (mean, SD)	54.81, 45.59	58.10, 44.53	0.266
QoL VT (mean, SD)	55.78, 22.31	63.29, 22.78	0.14
QoL MH (mean, SD)	58.04, 18.64	63.89, 22.26	0.21
QoL SF (mean, SD)	72.50, 23.32	74.29, 22.88	0.058
QoL BP (mean, SD)	70.67, 18.90	71.07, 16.94	0.92
QoL GH (mean, SD)	51.22, 21.46	53.29, 19.10	0.66
Sexual relationship (mean, SD)	65.42, 27.42	57.23, 24.85	0.17
Self-esteem (mean, SD)	66.81, 28.79	60.18, 26.95	0.30
Confidence (mean, SD)	67.41, 27.86	60.71, 24.90	0.27
Overall relationship (mean, SD)	68.61, 29.51	61.79, 26.94	0.29

### Potential risk factors for participants sexual dysfunction using logistic regression

Finally, we conducted a logistic regression analysis of potential risk factors for SD in all participants. Potential risk factors mainly include somking, drinking, mental work dominant quality of life indicators, Sexual relationship, Confidence, Self-esteem, age and BMI. In addition, we also analyze the relationship between SAPHO duration and SD. The specific data analysis was illustrated in Tables 5 and 6. The final results showed that whether it is male or female SD patients, the logistic regression data show that the above factors are not potential risk factors for sexual dysfunction. Similarly, SAPHO duration is not a potential risk factor for sexual dysfunction.

**Table 5:** Potential risk factors for female sexual dysfunction using logistic regression

Predictor		OR	95% CI	p
Current somker		0.153	0.022 ~ 1.053	0.06
Higher education		2.927	0.722 ~ 1.053	0.13
Mental work dominant		1.148	0.290 ~ 1.053	0.84
QoL Vitality		1.014	0.962 ~ 1.053	0.60
QoL Mental health		0.979	0.930 ~ 1.053	0.43
QoL Bodily pain		1.008	0.974 ~ 1.053	0.66
QoL General health		1.012	0.966 ~ 1.053	0.61
Sexual relationship		0.904	0.866 ~ 1.053	0.00*
Confidence		0.955	0.912 ~ 1.053	0.05
Current drinker		0.000	0.000 ~ 1.053	1.00
Age, years				
	18 ~ 29			
	30 ~ 39	0.412	0.008 ~ 1.053	0.66
	40 ~ 49	0.315	0.006 ~ 1.053	0.56
	50 ~ 59	0.268	0.005 ~ 1.053	0.52
SAPHO duration, years				
	< 5			
	5 ≤ to < 10	1.060	0.233 ~ 1.053	0.94
	≥ 10	1.178	0.171 ~ 1.053	0.87
BMI, kg/m^2^				
	< 18.5			
	18.5 ≤ to < 24	6.571	0.190 ~ 1.053	0.30
	24 ≤ to < 28	8.734	0.224 ~ 1.053	0.25
	≥ 28	34.573	0.535 ~ 1.053	0.10

**Table 6:** Potential risk factors for male erectile dysfunction using logistic regression

Predictor		OR	95% CI	p
BMI, kg/m^2^				
	< 18.5	Referent	Referent	Referent
	18.5 ≤ to < 24	0.000	0	1.00
	24 ≤ to < 28	0.000	0	1.00
QoL Physical functioning		0.986	0.953 ~ 1.021	0.44
QoL Vitality		0.974	0.937 ~ 1.021	0.20
QoL Social functioning		1.017	0.983 ~ 1.021	0.33
QoL Mental health		0.990	0.952 ~ 1.021	0.63
Sexual relationship		1.019	0.975 ~ 1.021	0.41
Self-esteem		0.995	0.918 ~ 1.021	0.91
Confidence		1.008	0.927 ~ 1.021	0.86

BMI, body mass index; PF, Physical-Functioning; RP, role-limit-physical; RE, role-limit-emotional; VT, energy-fatigue; MH, emotional-well-being; SF, social-functioning; BP, pain; GH, general-health

## Discussion

This study included 249 SAPHO syndrome patients, who were distributed across the country. Through the SF-36 QoL, FSFI, IIEF, and SEAR scales, we recorded the demographic, quality of life, quality of sexual life data and mental state of all participants (including 80 men and 169 women). Finally, we perform statistical analysis on all the data. In this article, we explored the connection between SAPHO and sexual dysfunction, and analyzed the potential risk factors that cause sexual dysfunction.

The incidence of sexual dysfunction is increasing year by year globally. SD affects the physical health of patients and reduces their quality of life. The cause of SD is unclear, but it may be multifactorial, involving biological, psychosocial, and disease-specific factors. SD is associated with many diseases, including IBD [15], chronic sinusitis [16], primary Sjögren’s syndrome (pSS) [17], rheumatoid arthritis (RA) [18], liver cirrhosis [19], diabetes [20], Behçet’s disease [11, 21] and so on. SAPHO syndrome is a chronic systemic inflammatory disease with external manifestations of skin involvement and internal manifestations of bone and joint involvement. The clinical presentation of the disease in SAPHO syndrome approximates that of seronegative arthritis. Since rheumatoid factor is negative in the early stages of rheumatoid arthritis, it can be called seronegative arthritis. Numerous studies have reported the relationship between rheumatoid arthritis and sexual dysfunction. Wojciech Tański et al. found that sexual dysfunction in patients with rheumatoid arthritis is closely related to their disease activity [22]. Patients with high disease activity had a higher proportion of sexual dysfunction. Interestingly, we found that SAPHO, as a disease similar to seronegative arthritis (early stage rheumatoid arthritis, low disease activity), also causes sexual dysfunction in patients. In this study, we found that out of 169 female patients, 124 patients (73.4%) had SD; among 80 male patients, 45 patients suffered from ED (56.3%); while 35 patients did not have ED (43.7%). Female patients suffer from SD more frequently than men. We then assessed the quality of life and mental state of all participants. We found that female SD patients had significantly lower scores in energy-fatigue, emotional-well-being, Sexual relationship, Self-esteem, Confidence and Overall relationship; among all male participants, we found that with or without SD There were no significant differences in the quality of life and mental state of the participants. This suggests that female SD patients are abnormal in their psychological state. It is worth noting that, with or without SD, there was no significant difference in the duration of SAPHO between female and male participants. Therefore, SAPHO duration will not have a significant impact on the patient’s SD.

The strength of this study is that this is the first report on the relationship between SAPHO syndrome and sexual dysfunction. At the same time, it included a relatively large sample size of 249 patients. Also, including 80 males is an advantage. However, the proportion of male participants was relatively low, and more male participants would make the conclusions more reliable. Study limitations include a significant risk of selection bias. Patients were consecutively recruited upon presentation to the outpatient department. Therefore, patients with low or very high disease activity may have been missed due to absence. Likewise, underresourced and noncompliant patients may also be spilled. However, this study did not classify the patients according to the site of disease involvement, which made it impossible for us to analyze the relationship between the site of disease involvement and sexual dysfunction in SAPHO patients. Sensitive questions about private and intimate matters involved in the questionnaire may make the experimental data of the survey research low quality. The cross-sectional study design did not allow for a dynamic lifetime approach [23]. In addition, since healthy participants were not included in this study, direct comparisons of sexual dysfunction data between healthy participants and patients with SAPHO syndrome could not be made. Therefore, a causal relationship between the various risk factors of SAPHO syndrome and sexual dysfunction cannot be drawn.

In conclusion, we found that the incidence of female SD in SAPHO patients is higher, and the main influence on female SD is psychological factors. This suggests that we are also very important to regulate the mental state of patients when treating SAPHO clinically. These promising findings prove that it is necessary to further design randomized controlled trials to further explore the relationship between SAPHO and SD.

## Data Availability

The datasets analyzed for this study are available from the corresponding author Dr. Chen Li (casio1981@163.com) upon reasonable request.
